# Comparison of the Predictive Ability of the Blood Urea Nitrogen/Albumin, C-Reactive Protein/Albumin, and Lactate/Albumin Ratios for Short-Term Mortality in SARS-CoV-2-Infected Patients

**DOI:** 10.1055/s-0043-1761471

**Published:** 2023-02-23

**Authors:** Serdar Özdemir, İbrahim Altunok

**Affiliations:** 1 Department of Emergency Medicine, Ümraniye Training and Research Hospital, University of Health Sciences, Istanbul, Turkey

**Keywords:** biomarkers, albumin, C-reactive protein, SARS-CoV-2, coronavirus disease 2019, blood urea nitrogen

## Abstract

**Background** Hematological parameters and their ratios are the most studied biomarkers for prediction of mortality or severe illness in severe acute respiratory syndrome coronavirus 2 (SARS-CoV-2) infection. This study aims to compare the power of the blood urea nitrogen (BUN)/albumin ratio, lactate/albumin ratio, and C-reactive protein (CRP)/albumin ratio, measured at the time of admission, in predicting 30-day mortality in SARS-CoV-2-infected patients presenting to the emergency department (ED).

**Materials and Methods** This retrospectively designed, single-center, observational study was performed in the ED of a tertiary education health care center. We documented the data of patients admitted with a confirmed SARS-CoV-2 infection between September 1, 2020, and January 1, 2021.

**Results** Of the 470 patients included in the study, 232 (49.4%) were female. The all-cause 30-day mortality rate was 23.8%. The area under the curve values for the BUN/albumin ratio, lactate/albumin ratio, and CRP/albumin ratio in the prediction of 30-day mortality were 0.725, 0.641, and 0.749, respectively. Sensitivity and negative predictive value for CRP/albumin ratio (≥0.049) and specificity for BUN/albumin ratio (≥1.17) were 92.86, 94.9, and 71.23, respectively. The odds ratio values of the BUN/albumin ratio (≥1.17), CRP/albumin ratio (≥0.049), and lactate/albumin ratio (≥0.046) for 30-day mortality were determined as 4.886, 9.268, and 2.518, respectively.

**Conclusion** The BUN/albumin ratio and CRP/albumin ratio can be used to predict 30-day mortality in SARS-CoV-2-infected patients admitted to ED. Furthermore, CRP/albumin ratio had the highest sensitivity and negative predictive value, while BUN/albumin ratio had the highest specificity.

## Introduction

Since coronavirus disease 2019 was first identified in December 2019, many researchers around the world have investigated the diagnosis, treatment, and prognostic factors of the disease. 1 The rapidly increasing number of patients placing an additional burden on the health system has made it necessary to predict the prognosis of this previously unknown disease. The limited number of hospital beds has increased the importance of identifying the critical patient and identifying patients in need of medical support. 2 Researchers are particularly working on easily accessible and cheaper biomarkers to detect critical illness.

Hematological parameters and their ratios are the most studied biomarkers for prediction of mortality or severe illness. 3 To increase the predictability of such tests, researchers have also examined the ratios of these parameters to each other. 3 4 5 The neutrophil/lymphocyte ratio has been reported to predict severe disease, intensive care need, and mortality among patients infected with SARS-CoV-2. 5 We hypothesized that the ratio of biochemical markers divided by albumin might be more reliable in predicting short-term mortality in these patients. Since albumin is a negative acute phase reactant and low albumin levels are associated with mortality in SARS-COV-2-infected patients, we think that dividing biochemical markers by albumin will increase the predictability of these parameters. In the current literature, blood urea nitrogen (BUN)/albumin ratio, lactate/albumin ratio, and C-reactive protein (CRP)/albumin ratio were tested as a predictor in SARS-CoV-2 infection.

In this study, we sought to compare the predictive power of the BUN/albumin ratio, lactate/albumin ratio, and CRP/albumin ratio, evaluated at the time of admission, in predicting all-cause 30-day mortality in SARS-CoV-2-infected patients presenting to the emergency department (ED) and reveal which is the best predictor.

## Materials and Methods

### Study Design

This retrospectively designed, single-center, observational study was performed at the Ümraniye Training and Research Hospital, University of Health Sciences, a tertiary education health care center with annual ED visits of 438,000 patients and a capacity of 682 beds. We documented the data of patients admitted to ED with a SARS-CoV-2 infection between September 1, 2020, and January 1, 2021.

### Study Population

**Fig. 1:**
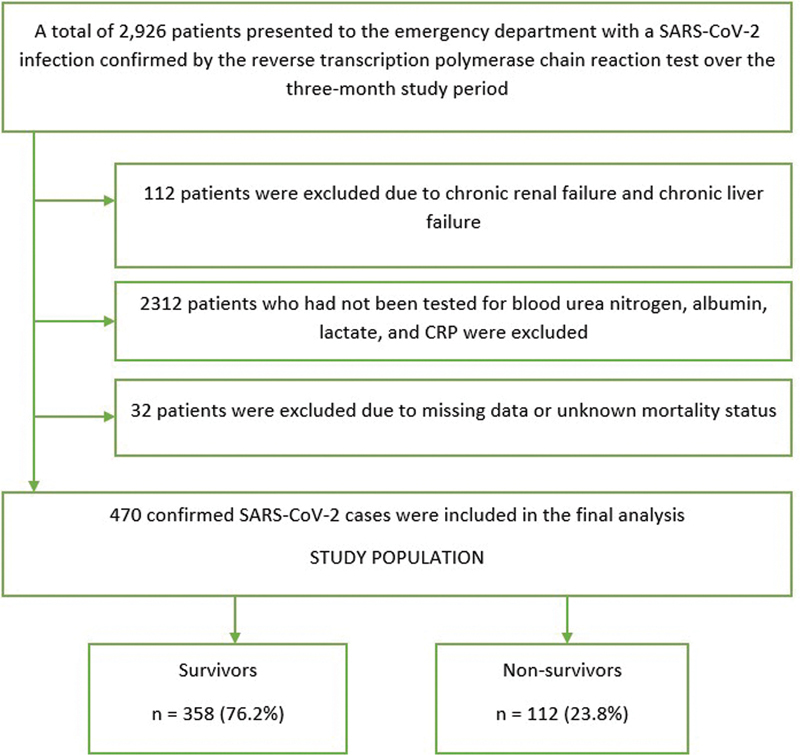
Flowchart of the study. CRP, C-reactive protein; SARS-CoV-2, severe acute respiratory syndrome coronavirus 2.

Our study population consisted of patients with a SARS-CoV-2 infection confirmed by the reverse transcription polymerase chain reaction (rt-PCR) test, who presented to our ED between September 1, 2020, and January 1, 2021. All the hospitalized patients and outpatients who had one positive result among the several rt-PCR tests performed were included in the study. Patients who had not been tested for BUN, lactate, CRP, and albumin were excluded from the study. Patients with chronic renal failure (since high baseline BUN levels) and chronic liver failure (since there might be weak CRP response and low baseline albumin values) were also excluded. The flowchart of the study is shown in Fig. 1 .

### Data Collection

The data of patients that presented to our ED and had at least one positive rt-PCR result for SARS-CoV-2 were obtained from the hospital computer-based information system. The comorbidities, laboratory parameters, and death information of the patients were recorded. The comorbidities were noted as chronic obstructive pulmonary diseases, hypertension, diabetes mellitus, coronary artery disease, congestive heart failure, history of malignancy, hyperlipidemia, and history of cerebrovascular disease. Patients with chronic kidney disease and chronic liver disease were excluded. White blood cell count, neutrophil count, lymphocyte count, platelet count, mean platelet volume, plateletcrit, BUN, lactate, CRP, and albumin were recorded. The all-cause 30-day mortality data of all patients were obtained using the national death notification system, where death information for all patients is legally accessible.

### Statistical Analysis

SPSS version 22.0 for Windows (SPSS Inc, Chicago, Illinois, United States) was used to perform all statistical analyses. The normality analysis of data was conducted using the Kolmogorov–Smirnov's test. Categorical data were compared using the chi-square test. Quantitative variables were presented as interquartile range (25th–75th percentiles) and median values. Quantitative variables were compared using the Mann–Whitney's test or Student's *t* -test according to the normality of distribution for the two groups. The Bonferroni correction was used as a method to counteract the problem of multiple comparisons. To identify variables associated with 30-day mortality status, the univariate analysis was undertaken using the chi-square test, Fisher's exact test, Student's *t* -test, and Mann–Whitney's *U* test, where appropriate. The receiver operating characteristic (ROC) curves were used to assess the accuracy of parameters to predict mortality, and the results were reported as the area under the curve (AUC) values. Youden's index was obtained to determine the optimal cutoff value for scores with the highest sensitivity and specificity.

The AUC, 95% confidence interval (CI), cutoff, sensitivity, specificity, positive predictive value, and negative predictive value of all parameters are presented in Table 1 . The AUC values more than 0.7 were accepted as predictive of mortality. 6 The comparison of the AUC values was made with the DeLong's equality test. We have prepared a two-by-two frequency table according to the optimal cutoff values of ratios determined according to Youden's index and mortality data. Odds ratios (OR) were calculated by using two-by-two frequency tables of ratios. 7 ORs were used to compare the ability of the parameters to predict mortality. Statistical significance was defined at *p* < 0.05.

**Table 1:** Accuracy of ratios measured at the time of emergency department admission in predicting 30-day all-cause mortality

	AUC	Cutoff	Sensitivity	Specificity	PPV	NPV	LR+	LR−	*p* -Value
C-reactive protein/albumin ratio	0.749	≥0.049	92.86	41.62	33.23	94.90	1.59	0.17	**<0.001**
Blood urea nitrogen/albumin ratio	0.725	≥1.17	66.96	71.23	42.13	87.33	2.33	0.46	**<0.001**
Lactate/albumin ratio	0.641	≥0.046	56.25	66.48	34.43	82.93	1.68	0.66	**<0.001**

Abbreviations: AUC, area under the curve; LR, Likelihood ratio; NPV, negative predictive value; PPV, positive predictive value.

### Ethics

Ethical approval was obtained from the local clinical research ethics committee with the approval number B.10. 1.TKH.4.34. H. GP.0.01/17. We retrospectively reviewed the data extracted from the computer-based hospital information management system. The extracted data were solely clinical and did not include any personal or identifiable information. Therefore, the necessity for informed consent was waived.

## Results

Of the 470 patients included in the study, 232 (49.4%) were female. The median age of the 470 patients was 69 (25th–75th percentiles: 54.75–79) years. A total of 112 patients died within 30 days of ED presentation. The mortality rate of all-cause 30-day mortality was 23.8% for our study cohort. Table 2 presents the demographic characteristics, comorbid diseases, and laboratory parameters and the comparison of these variables between the survivor and nonsurvivor groups.

**Table 2:** Baseline characteristics and laboratory parameters of the enrolled patients and their comparison between the survivor and nonsurvivor groups

Variables	Total *n* = 470	Survivor *n* = 358 (76.2%)	Nonsurvivor *n* = 112 (23.8%)	*p* -Values
	*n* (%)/median (25th–75th percentiles)	*n* (%)/median (25th–75th percentiles)	*n* (%)/median (25th–75th percentiles)	
Age	69 (54.75–79)	64.5 (50–75.25)	79 (70.25–86)	**<0.001**
< 65 y	195 (41.5%)	179 (50%)	16 (14.3%)	**<0.001**
≥65 y	275 (58.5%)	179 (50%9	96 (85.7%)	
Gender				0.421
Female	238 (50.6%)	185 (51.7%)	53 (47.3%)	
Male	232 (49.4%)	173 (48.3%)	59 (52.7%)	
Comorbidities				
Chronic obstructive pulmonary disease	61 (13%)	41 (67.2%)	20 (32.8%)	0.078
Hypertension	261(55.5%)	183 (70.1%)	78 (29.9%)	**<0.001**
Diabetes mellitus	151 (32.1%)	106 (70.2%)	45 (29.8%)	0.037
Coronary artery disease	120 (25.5)	80 (66.7%)	40 (33.3%)	0.005
Congestive heart failure	41 (8.7%)	25 (61%)	16 (39%)	0.017
History of malignancy	33 (7%)	22 (66.7%)	11 (33.3%)	0.184
Hyperlipidemia	162 (34.5%)	108 (66.7%)	54 (33.3%)	**<0.001**
History of cerebrovascular disease	42 (8.9%)	24 (57.1%)	18 (42.9%)	0.002
Laboratory parameters				
White blood cell count (/µL)	6.24 (4.77–8.08)	6.04 (4.75–7.71)	7.39 (4.85–10.13)	**<0.001**
Neutrophil count (/µL)	4.27 (3.12–6.17)	4.02 (2.96–5.82)	5.56 (3.67–8.00)	**<0.001**
Lymphocyte count (/µL)	1.17 (0.83–1.65)	1.24 (0.89–1.75)	0.92 (0.67–1.34)	**<0.001**
Platelet count (103/µL)	191.5 (154–247)	196 (156–149)	184.5 (148.5–227)	0.156
Mean platelet volume (fL)	9.75 (9.00–10.60)	9.70 (9.00–10.63)	9.85 (9.00–10.60)	0.718
Plateletcrit (%)	0.19 (0.15–0.249	0.19 (0.16–0.24)	0.18 (0.14–0.24)	0.291
Blood urea nitrogen (mg/dL)	38.52 (27.82–55.64)	34.24 (25.68–47.62)	53.50 (36.38–32.93)	**<0.001**
Lactate (mmol/L)	1.6 (1.2–2.0)	1.5 (1.1–2)	1.8 (1.3–2.3)	**0.002**
C-reactive protein, (mg/dL)	4.3 (1.1–9.2)	3.3 (0.7–7.2)	8.8 (4.3–16.2)	**<0.001**
Albumin (g/dL)	37.8 (34.6–40.7)	38.6 (37.6–41.4)	35.2 (32.8–37.7)	**<0.001**
Neutrophil/lymphocyte ratio	3.32 (2.27–6.46)	2.96 (2.10–5.38)	5.38 (2.93–9.46)	**<0.001**
Platelet/lymphocyte ratio	161.44 (116.20–234.39)	154.85 (11.55–220.88)	195.53 (128.82–125.07)	**<0.001**
C-reactive protein/albumin ratio	0.112 (0.026–0.266)	0.080 (0.018–0.197)	0.262 (0.112–0.470)	**<0.001**
Blood urea nitrogen/albumin ratio	1.02 (0.70–1.59)	0.92 (0.65–1.34)	1.49 (0.98–2.48)	**<0.001**
Lactate/albumin ratio	0.041 (0.031–0.055)	0.040 (0.031–0.051)	0.049 (0.037–0.067)	**<0.001**

Note: Bonferroni-corrected *p* -value: 0.0019.

**Fig. 2:**
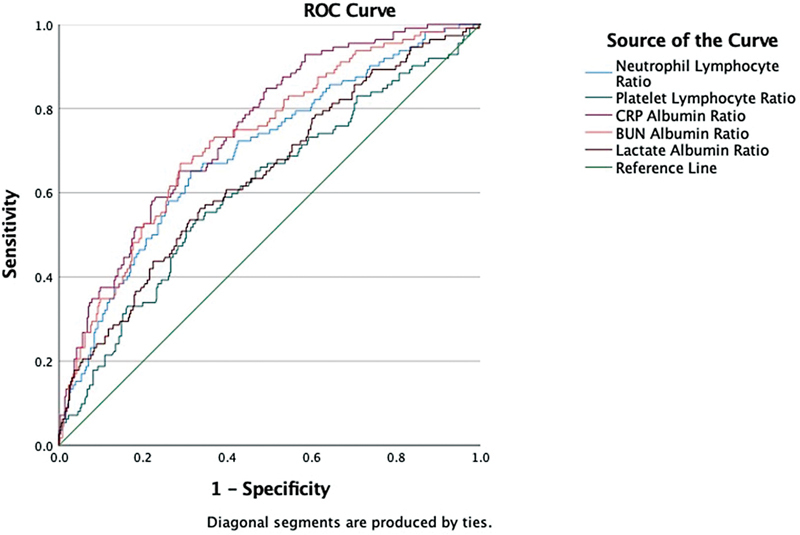
Receiver operating characteristic (ROC) curves for the neutrophil/lymphocyte ratio, platelet/lymphocyte ratio, C-reactive protein (CRP)/albumin ratio, blood urea nitrogen (BUN)/albumin ratio, and lactate/albumin ratio for the prediction of 30-day mortality in patients with severe acute respiratory syndrome coronavirus 2 infection.

The ROC curve analysis was performed to determine the discriminative ability of laboratory parameters for 30-day mortality. Table 1 and Fig. 2 present the cutoff values of BUN/albumin ratio, CRP/albumin ratio, and lactate/albumin ratio according to the best Youden's index, as well as their sensitivity, specificity, likelihood ratio, and AUC values. According to the best Youden's index, the cutoff and AUC values were determined as 0.049 (sensitivity: 92.86%, specificity: 41.62%) and 0.749, respectively, for CRP/albumin ratio; 1.17 (sensitivity: 66.96%, specificity: 71.23%) and 0.725, respectively, for BUN/albumin ratio; and 0.046 (sensitivity: 56.25%, specificity: 66.48%) and 0.641, respectively, for lactate/albumin ratio. There was no statistically significant difference between the AUC values of CRP/albumin ratio and BUN/albumin ratio (0.749 and 0.725, respectively, *p* = 0.425, DeLong's equality test). The AUC values of CRP/albumin ratio and BUN/albumin ratio (0.749 and 0.725, respectively) were significantly higher than the AUC value of lactate/albumin ratio (0.641) ( *p* = 0.002 for CRP/albumin ratio vs. lactate/albumin ratio and *p* = 0.021 for BUN/albumin ratio vs. lactate/albumin ratio, DeLong's equality test).

The OR values of BUN/albumin ratio (≥1.17), CRP/albumin ratio (≥0.049), and lactate/albumin ratio (≥0.046) for 30-day mortality were determined as 4.886 (95% CI: 3.108–7.682), 9.268 (95% CI: 4.381–19.605), and 2.518 (95% CI: 1.634–3.882), respectively.

## Discussion

Many scoring systems and laboratory parameters have been studied to assess mortality and severity in SARS-CoV-2-infected patients. Identifying an ideal predictor is necessary for the optimum use of the facilities of hospitals and the health system. In the current study, we compared three ratios formed with albumin in predicting 30-day mortality. Our analyses determined that although CRP/albumin ratio had the highest sensitivity and negative predictive value and BUN/albumin ratio had the highest specificity, BUN/albumin ratio and CRP/albumin ratio had similar ability to predict all-cause 30-day mortality in patients with SARS-CoV-2 infection, while lactate/albumin ratio did not have predictive power. To our knowledge, our study is the first to compare the ability of BUN/albumin ratio, CRP/albumin ratio, and lactate/albumin ratio to predict short-term mortality in patients presenting to the ED with a SARS-CoV-2 infection.

The predictive power of BUN/albumin ratio has been investigated in many different patient groups presenting to ED. 8 Since the beginning of the pandemic, researchers have conducted studies revealing the importance of BUN/albumin ratio in patients with SARS-CoV-2 infection. 9 10 In a study conducted in ED with 600 patients, Küçükceran et al tested the predictive ability of BUN/albumin ratio for in-hospital mortality. They reported that BUN/albumin ratio could be used as a predictive parameter with an AUC value of 0.809 and OR value of 10.44. 9 In another study, Huang et al tested the predictive power of BUN/albumin ratio for severe disease in SARS-CoV-2 infection. The authors reported that an elevated BUN/albumin ratio level at admission was an independent risk factor for severe disease and could be used as a predictor with an AUC value of 0.821. 10 The results of our study, in line with the literature, revealed that BUN/albumin ratio might be a predictor of all-cause 30-day mortality in ED with highest specificity.

CRP/albumin ratio has been investigated as a predictor in many diseases, 11 and similar to BUN/albumin ratio, it has been determined as a parameter associated with mortality in SARS-CoV-2-infected patients in the current literature. 12 13 Güney et al demonstrated that BUN/albumin ratio is an independent predictor of in-hospital mortality in patients hospitalized due to SARS-CoV-2 infection. 12 Torun et al showed that CRP/albumin ratio, fibrinogen/albumin ratio, and neutrophil/lymphocyte ratio were predictors for severe disease in SARS-CoV-2-infected patients. In addition, they reported that CRP/albumin ratio was the best predictor of severe SARS-CoV-2 infection when compared with fibrinogen/albumin ratio and neutrophil/lymphocyte ratio, based on the results of their cohort. 13 Demir et al reported a correlation between CRP/albumin ratio and computed tomography findings in patients with a SARS-CoV-2 infection confirmed by an rt-PCR test. 14 Similar to these studies, the analysis results of our study also revealed a relationship between CRP/albumin ratio and mortality, suggesting that CRP/albumin ratio could be used as a predictor of mortality in SARS-CoV-2-infected patients with highest sensitivity and negative predictive value.

Although lactate/albumin ratio is a well-known predictor in critical and septic patients, 15 it is the least researched ratio for SARS-CoV-2-infected patients among the ratios investigated in our study. The Korean Shock Society investigators evaluated the prognostic value of lactate/albumin ratio in patients with critical sepsis in a multicenter study and found it to be a good predictor of critical illness with a low AUC value of 0.68. 16 Gök et al, in an intensive care study in which they investigated the relationship between lactate/albumin ratio and short-term mortality, showed that lactate/albumin ratio could be used as a prognostic factor in SARS-CoV-2-infected patients with an AUC value of 0.824. 17 Although there was a difference between the mortality and survivor groups in terms of lactate/albumin ratio as a result of the univariate test, lactate/albumin ratio had the lowest AUC value of 0.641 in the ROC analysis, indicating that it could not be accepted as a good predictor. 6

The first and main limitation of our study was that it was designed retrospectively. There may have been other risk factors that could not be measured due to the retrospective design. In addition, we only included patients who had been tested for parameters that we investigated. Patients who were not tested by the clinician for these parameters, especially blood gas analysis for lactate, were not included in the study. This may have caused our study population to include more severe patients. Furthermore, there was a significant difference between the deceased and survivor groups in our sample in terms of age, hypertension, and hyperlipidemia. Although we do not think that this affected our results, we recommend repeating the study with more homogeneous groups. Finally, our study had a single-center design, and therefore, our results cannot be generalized to other health care institutions.

## Conclusion

In conclusion, the results of our study show that BUN/albumin ratio and CRP/albumin ratio can be used to predict 30-day mortality in SARS-CoV-2-infected patients admitted to ED. Furthermore, CRP/albumin ratio had the highest sensitivity and negative predictive value, while BUN/albumin ratio had the highest specificity.
